# Supplementary material for: Memory CD8 T cells are vulnerable to chronic IFN-γ signals but not to CD4 T cell deficiency in MHCII-deficient mice
Source: Nat Commun. 2024 May 28;15:4418. doi: 10.1038/s41467-024-48704-4 (PMC11133459; doi:10.1038/s41467-024-48704-4)
Supplement: Supplementary file 1 — Supplementary Information [file 41467_2024_48704_MOESM1_ESM.pdf]

Inventory of Supplementary Information

Supplementary Fig. 1 | Characterization of memory P14 T cells in MHCII$^{-/-}$, CD4$^{-/-}$, GK1.5-treated or non-treated secondary hosts

Supplementary Fig. 2 | IL-7 and IL-15 levels and phenotypes of memory P14 T cells

Supplementary Fig. 3 | Effects of rmIFN-γ on memory CD8 T cells *in vivo* and *in vitro*

Supplementary Fig. 4 | Ectopic expression of STAT1 and its mutant in CD8 T cells

Supplementary Fig. 5 | scRNA-seq on memory CD8 T cells from MHCII$^{-/-}$ and WT host mice

Supplementary Fig. 6 | The number of different lymphocyte subsets in MHCII$^{-/-}$ or WT mice

Supplementary Fig. 7 | Gating strategy

Supplementary Table 1 | Differentially expressed genes in memory P14 CD8 T cells from MHCII$^{-/-}$ host mice

Supplementary Table 2 | The 30 most differentially expressed genes in memory P14 CD8 T cell clusters

Supplementary Fig. 1

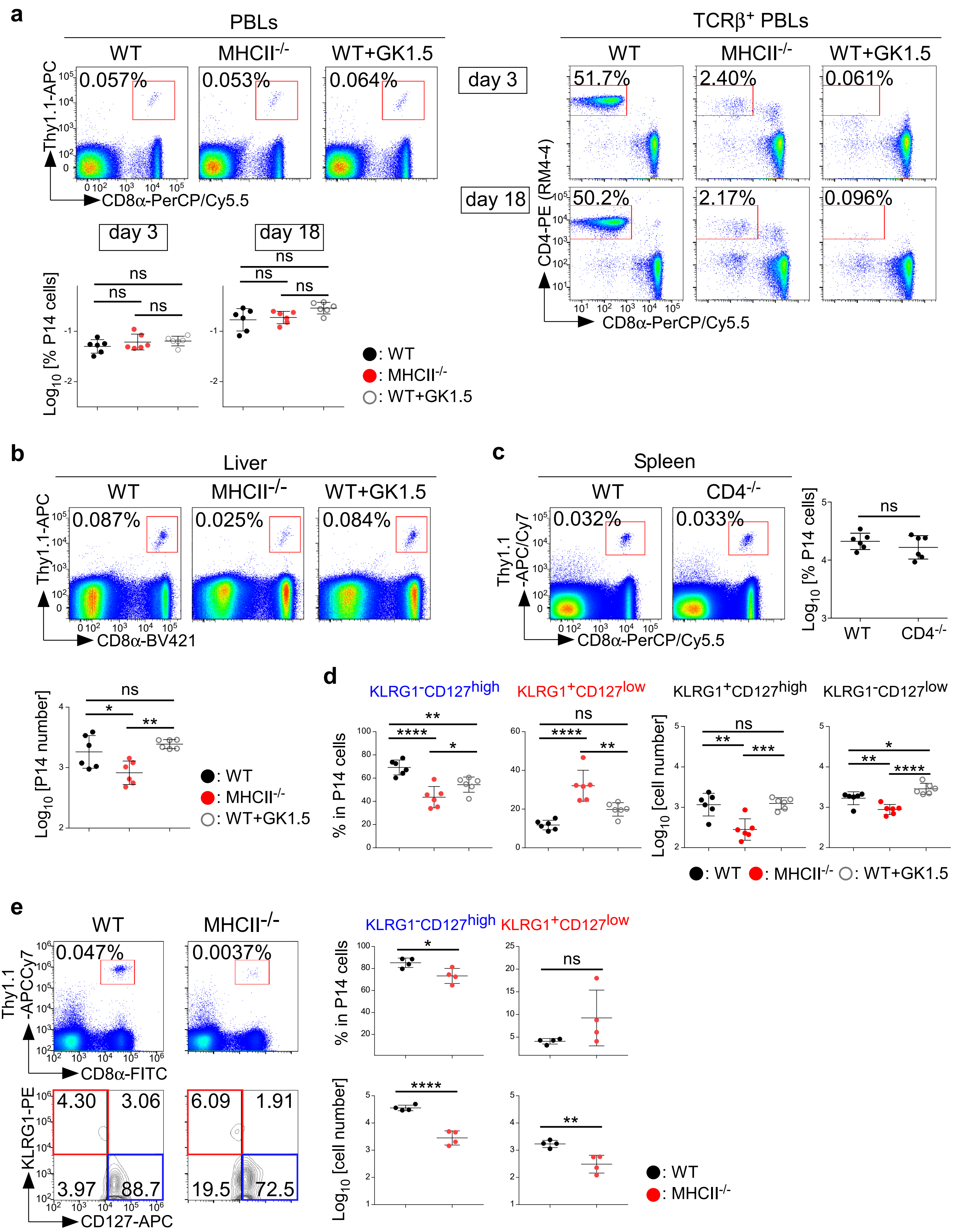

Supplementary Fig. 1 | Characterization of memory P14 T cells in MHCII$^{-/-}$, CD4$^{-/-}$, GK1.5-treated or non-treated secondary hosts

Thy1.1 memory P14 T cells were generated and transferred into Thy1.2 WT mice, MHCII$^{-/-}$ mice or WT mice treated with anti-CD4 mAb (GK1.5) or CD4$^{-/-}$ mice as described in Fig. 1a.

a Representative flow cytometric profiles of total PBLs on day 3 (left panels) and TCRβ^{+} PBLs on day3 and 18 (right panels). Percentages of Thy1.1^{+}CD8α^{+}Vα2^{+} P14 cells on day 3 or 18 after the secondary transfer are summarized (mean ±SD).

b Representative flow cytometric profiles of liver mononuclear leukocytes on days 40-50 after the secondary transfer. The number of Thy1.1^{+}CD8α^{+}Vα2^{+} P14 T cells is summarized (mean ±SD).

c Representative flow cytometric profiles of splenocytes from WT and CD4$^{-/-}$ mice on days 41-42 after the secondary transfer. The number of Thy1.1^{+}CD8α^{+}Vα2^{+} P14 T cells is summarized (mean ±SD).

d Percentages of indicated subsets in Thy1.1^{+}CD8α^{+}Vα2^{+} P14 T cells and the number of indicated subsets from the spleen on days 40-50 after the secondary transfer are summarized (mean ±SD).

e Representative flow cytometric profiles of total cells (upper left panels) and Thy1.1^{+}CD8α^{+}Vα2^{+} P14 T cells (lower left panels) from spleens on day 97 or 113 after the secondary transfer into WT and MHCII$^{-/-}$ mice. Percentages of indicated subsets in Thy1.1^{+}CD8α^{+}Vα2^{+} P14 T cells and the number of each subset are summarized on the right (mean ±SD).

Each symbol represents one mouse. Results are pooled from two independent experiments. Data were analyzed using one-way ANOVA with Tukey's multiple comparisons test (**a, b, d**) or unpaired *t*-test (**c, e**). Source data are provided as a Source Data file.

Supplementary Fig. 2

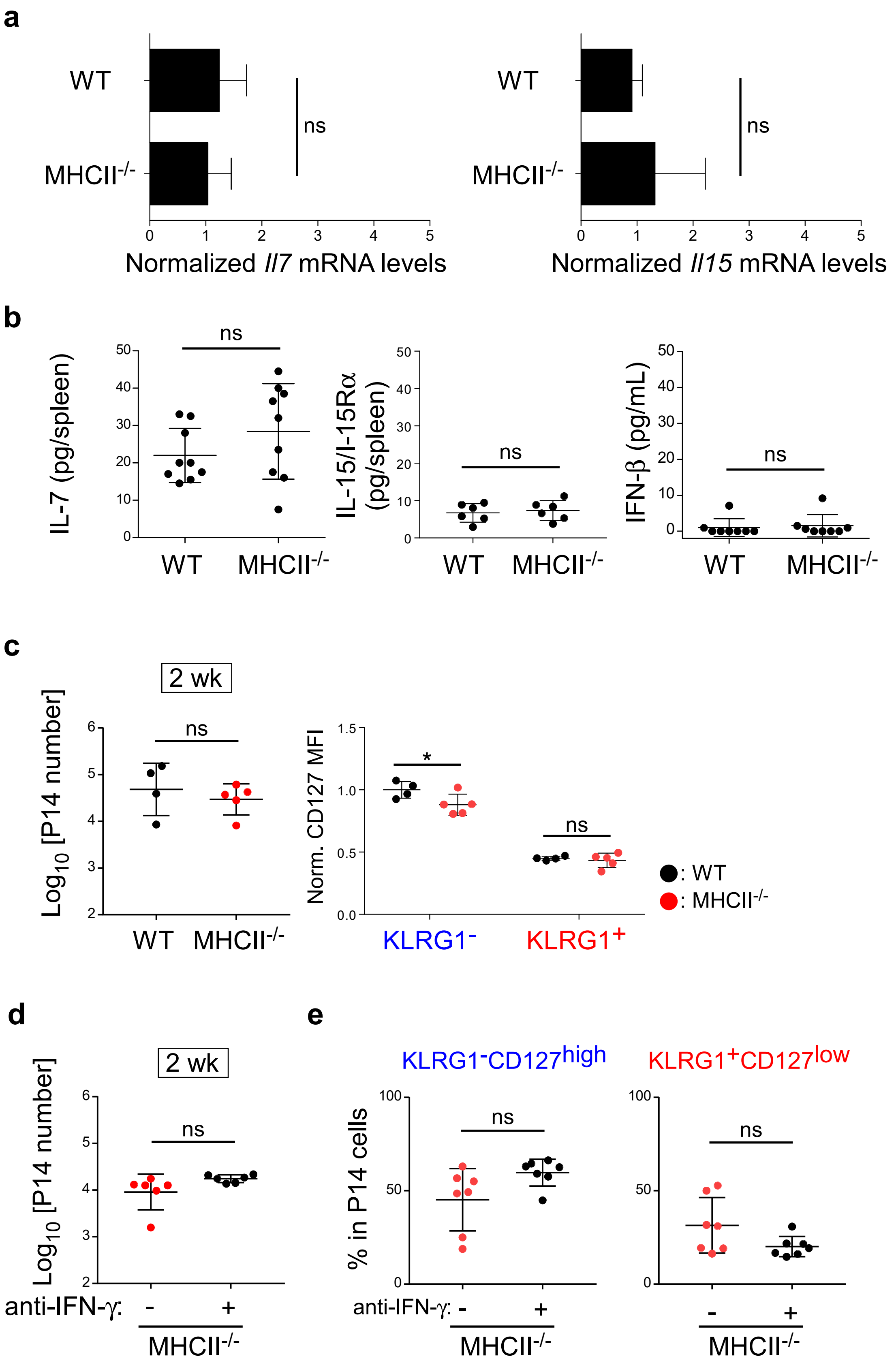

Supplementary Fig. 2 | IL-7 and IL-15 levels and phenotypes of memory P14 T cells

a *Il7* and *Il15* mRNA levels relative to *Hprt* mRNA levels in spleens (mean ±SD, n = 4 per group).

b Concentrations of IL-7 and the IL-15/IL-15Rα complex in splenic interstitial fluid, and IFN-β in plasma (mean ±SD).

c-e Thy1.1 memory P14 T cells were generated, transferred into MHCII$^{-/-}$, WT, or MHCII$^{-/-}$ mice treated with or without anti-IFN-γ mAb as in Fig. 2d-h.

c The number of Thy1.1^{+}CD8α^{+}Vα2^{+} P14 T cells from spleens on days 14-15 after transfer (mean ±SD) (left) and geometric MFI values of CD127 normalized by the average MFI values of KLRG1^{-} cells from WT mice are summarized (mean ±SD) (right).

d The number of Thy1.1^{+}CD8α^{+}Vα2^{+} P14 T cells from spleens on days 14-15 after transfer.

e Percentages of indicated subsets in Thy1.1^{+}CD8α^{+}Vα2^{+} P14 T cells from spleens are analyzed 40-50 days later and summarized (mean ±SD).

Each symbol indicates one mouse. Data were analyzed using unpaired *t*-tests or two-way ANOVA with Sidak's multiple comparisons test (c, right). Source data are provided as a Source Data file.

Supplementary Fig. 3

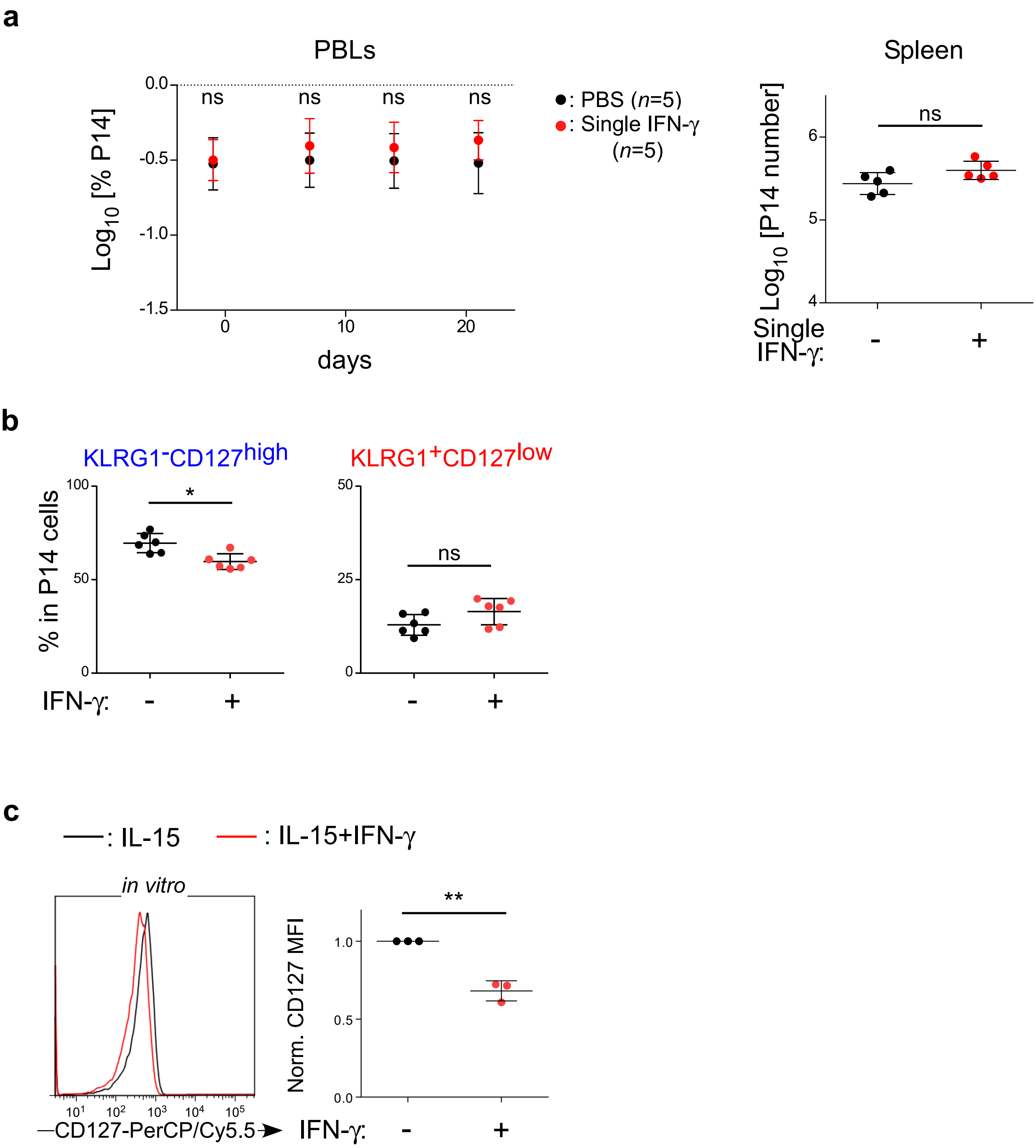

Supplementary Fig. 3 | Effects of rmIFN-γ on memory CD8 T cells *in vivo* and *in vitro*

a, b Thy1.1 naïve P14 T cells were transferred into Thy1.2 WT mice and infected with Vac-GP33, as in Fig. 1a.

a More than 30 days after infection, host mice received a single injection of rmIFN-γ or PBS. The time-course of the percentage of $Thy1.1^{+}CD8\alpha^{+}V\alpha2^{+}$ P14 T cells in PBLs (mean ±SD) following rmIFN-γ injection (left) and their number in the spleen on day 34 after the injection (right) are shown.

b More than 30 days after infection, host mice received repeated injections (3 times per week) of rmIFN-γ or PBS, as in Fig. 3. Percentages of indicated subsets in $Thy1.1^{+}CD8\alpha^{+}V\alpha2^{+}$ P14 T cells from spleens are summarized (mean ±SD).

c Two months after infection, $Thy1.1^{+}CD8\alpha^{+}$ memory P14 T cells were sorted from Thy1.2 WT host mice and cultured with or without IFN-γ in the presence of IL-15 for 7-8 days. Representative flow cytometric profiles of cultured P14 T cells and quantification of normalized geometric MFI values of CD127 are shown.

Data were analyzed using two-way ANOVA with Sidak's multiple comparisons test (left graph of **a**) or unpaired *t*-test (other graphs). Source data are provided as a Source Data file.

Supplementary Fig. 4

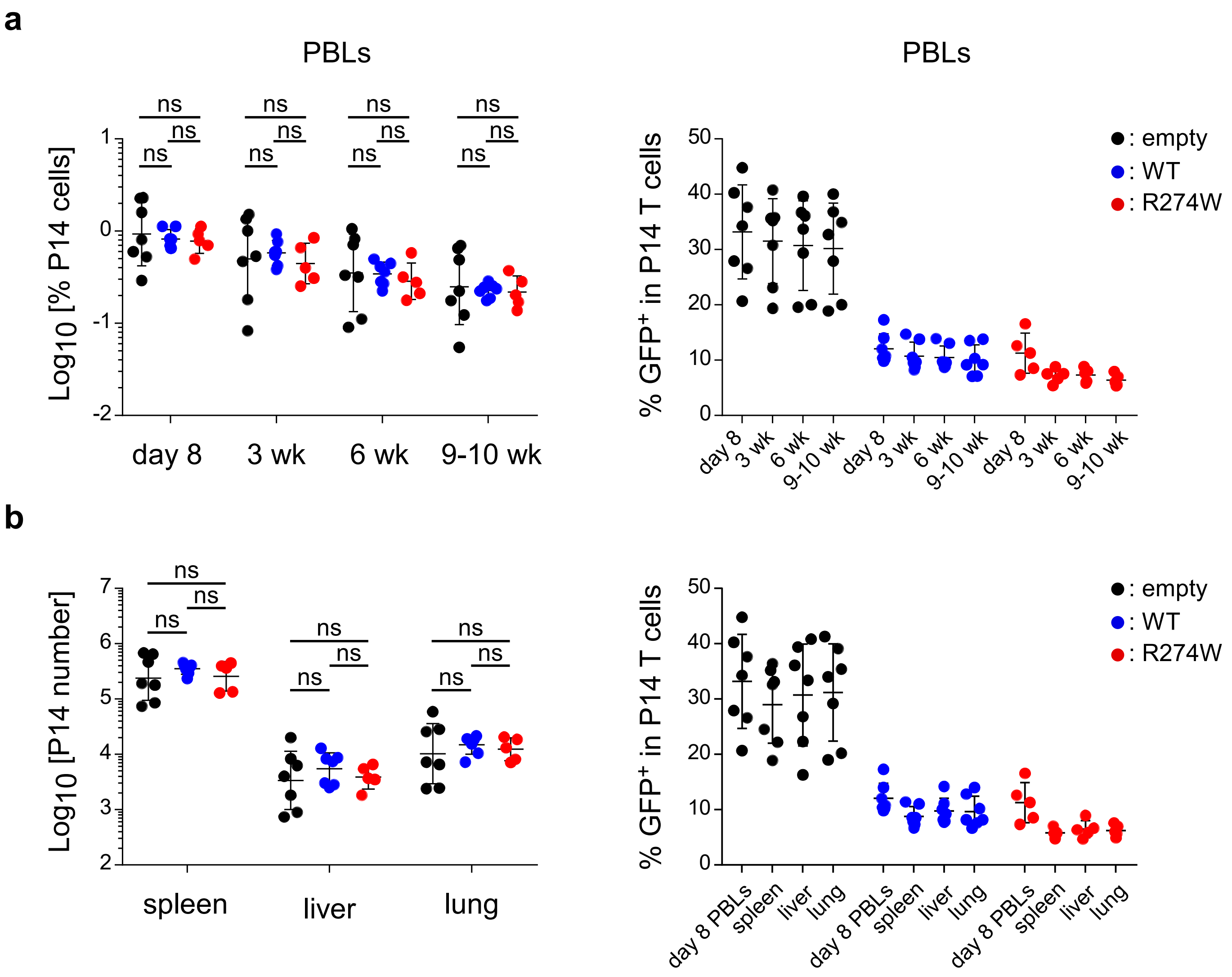

Supplementary Fig. 4 | Ectopic expression of STAT1 and its mutant in CD8 T cells

Thy1.1 naive P14 T cells were activated and transduced with retrovirus expressing mouse $STAT1^{WT}$, $STAT1^{R247W}$ or empty retrovirus and transferred into mice infected with Vac-GP33 one day earlier, as in Fig. 4.

a Percentages of $Thy1.1^{+}CD8\alpha^{+}V\alpha2^{+}$ P14 T cells in PBLs (left) and of GFP^{+} cells in $Thy1.1^{+}CD8\alpha^{+}V\alpha2^{+}$ PBLs (right) at the indicated times.

b The number of $Thy1.1^{+}CD8\alpha^{+}V\alpha2^{+}$ P14 T cells (left) and the percentage of GFP^{+} cells in $Thy1.1^{+}CD8\alpha^{+}V\alpha2^{+}$ P14 T cells from indicated organs 10-11 weeks after the infection (right).

Each symbol indicates one mouse. Results are pooled from two independent experiments. Data were analyzed using two-way ANOVA with Turkey's multiple comparisons test. Source data are provided as a Source Data file.

Supplementary Fig. 5

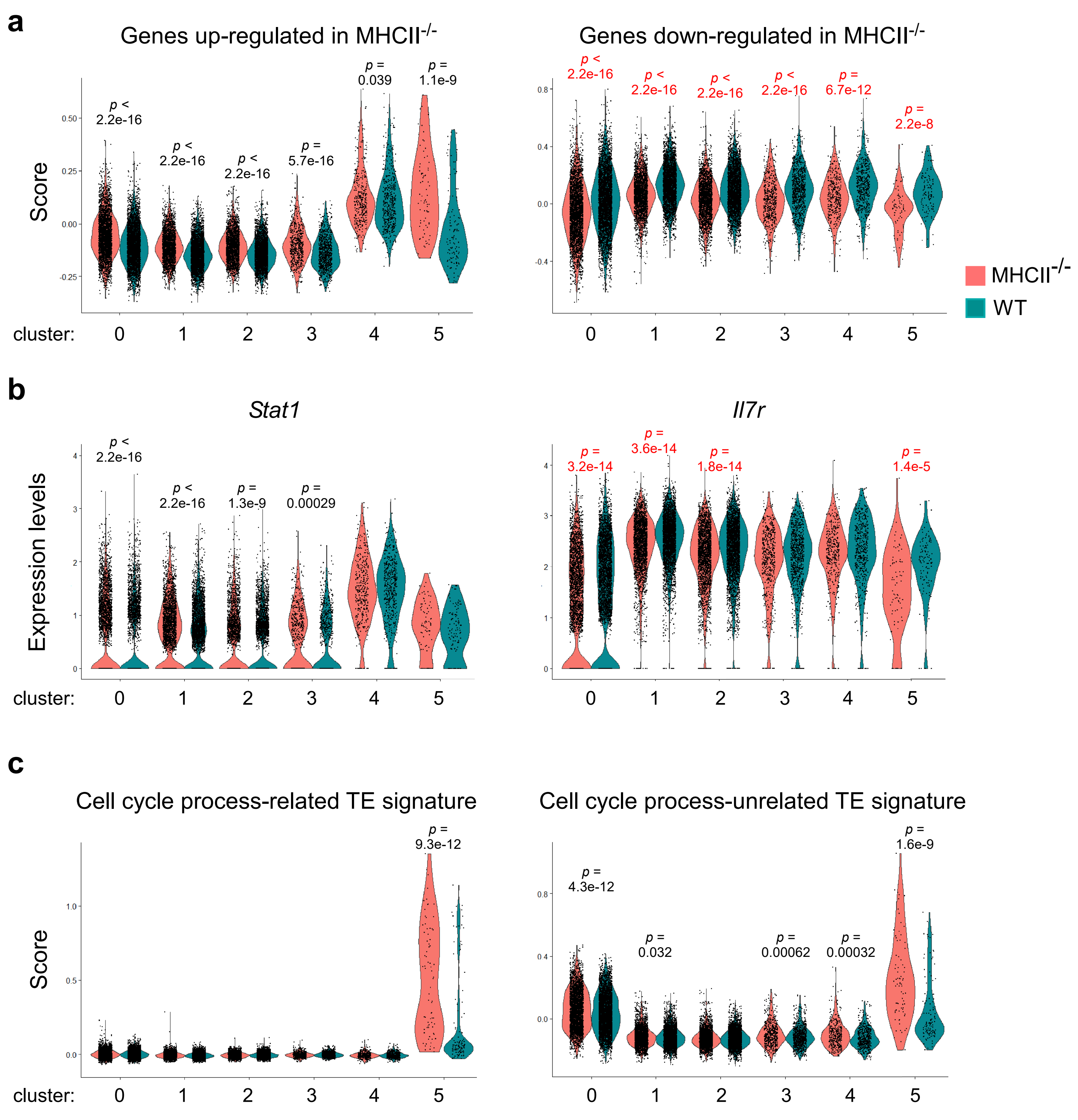

Supplementary Fig. 5 | scRNA-seq on memory CD8 T cells from MHCII$^{-/-}$ and WT host mice

a Relative expression levels of DEGs determined by bulk RNA-seq analysis upregulated (left) or downregulated (right) in memory P14 T cells from MHCII$^{-/-}$ mice relative to those from WT mice.

b Relative expression levels of *Stat1* and *Il7r*.

c Relative expression levels of TE cell signature genes that belong to the GO term "cell cycle process" (GO:0022402) (left) and those that do not (right).

P-values were calculated with the Wilcoxon rank-sum test. When the average of the MHCII$^{-/-}$ group is lower than that of the WT group, the *p*-value is indicated in red.

Supplementary Fig. 6

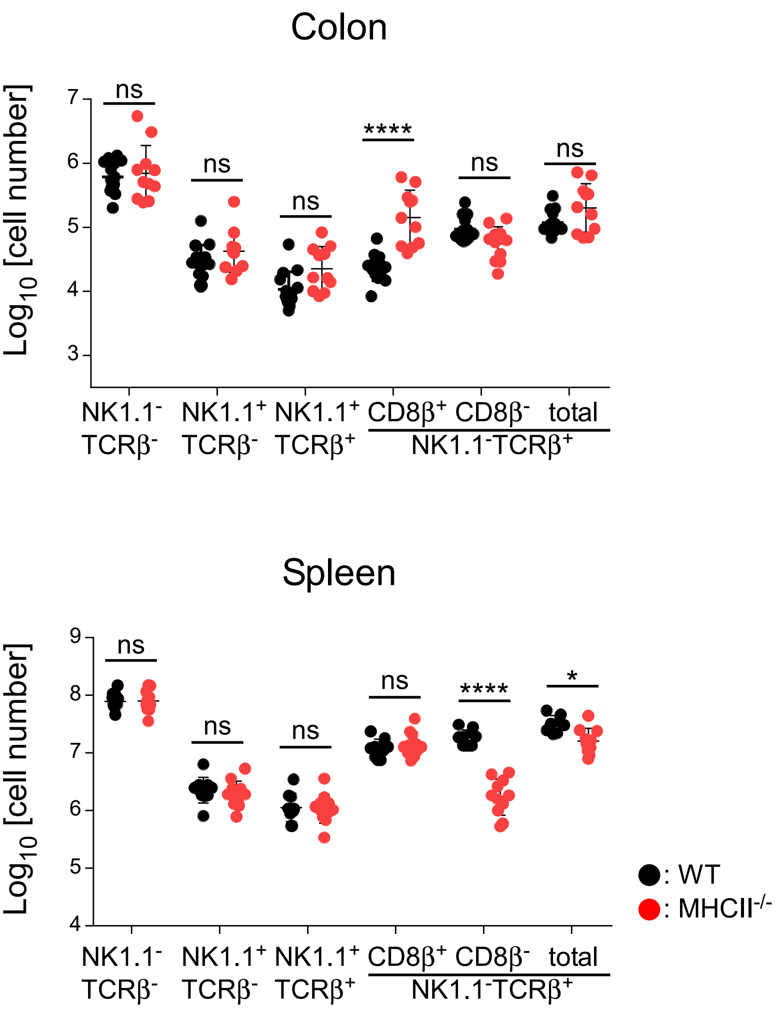

Supplementary Fig. 6 | The number of different lymphocyte subsets in MHCII$^{-/-}$ or WT mice

Colonic and splenic lymphocytes from IFN-γ Venus MHCII$^{-/-}$ or MHCII$^{+/-}$ (WT) mice were counted and analyzed by flow cytometry. The number of indicated subsets is summarized (mean ±SD). Data were analyzed using two-way ANOVA with Sidak's multiple comparisons test. Source data are provided as a Source Data file.

Supplementary Fig. 7

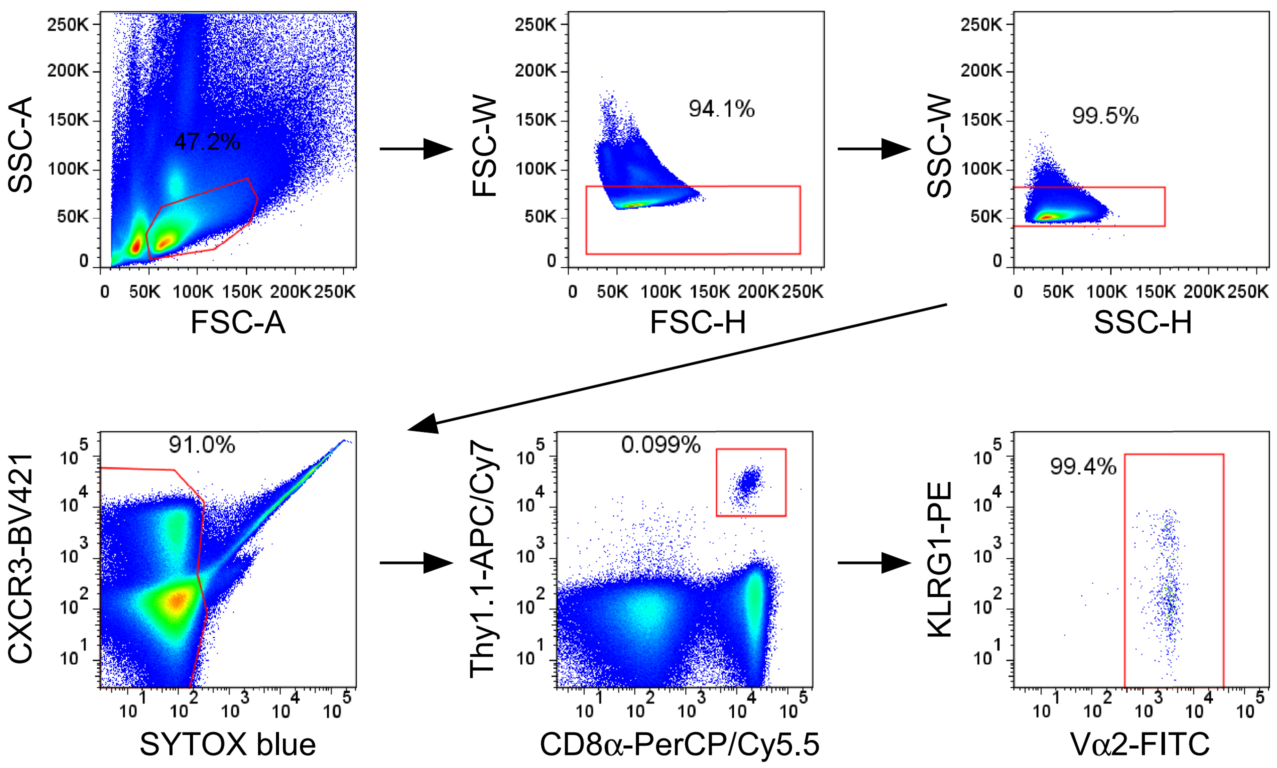

Supplementary Fig. 7 | Gating strategy

Gating strategy to define donor P14 T cells is shown. To examine expression of KLRG1 and memory T cell-associated markers (e.g., CD27, CD127, CXCR3), spleen cells were first depleted of B cells and adherent cells by panning and stained for these markers along with Thy1.1, CD8α, and Vα2.

Supplementary Table 1: Differentially expressed genes in memory P14 CD8 T cells from MHCII$^{-/-}$ host mice

P-values were calculated using the Wald test and corrected for multiple testing using the Benjamini-Hochberg procedure to obtain FDR values.

GeneName	baseMean	log2FoldChange: MHCII-/- vs. WT	lfcSE	*p*-value	FDR
Stat1	17494.36177	0.681935207	0.07892473	2.96E-19	3.53E-15
Dtx1	9664.1277	-0.74422135	0.092146448	3.35E-17	2.00E-13
Rrm2	1034.387975	1.738131324	0.226978096	8.97E-16	3.57E-12
Cd74	725.9432715	2.572574353	0.348360849	2.42E-15	5.78E-12
Rtp4	3207.969219	1.074212738	0.142347932	2.07E-15	5.78E-12
Mki67	836.24962	1.927977761	0.275527003	8.70E-14	1.49E-10
Igtp	6351.589166	0.750957531	0.106166245	7.63E-14	1.49E-10
Lilr4b	2640.545116	1.080709357	0.154021878	1.04E-13	1.56E-10
Ifit3	3896.553051	0.951256798	0.140424082	5.50E-13	7.30E-10
Gzma	9400.670262	0.627927619	0.092914631	7.29E-13	8.71E-10
Ly6a	8274.766224	0.694487841	0.104615166	1.54E-12	1.67E-09
Gzmb	6490.416398	0.970096652	0.148174849	2.00E-12	1.99E-09
Il7r	36531.9732	-0.523943899	0.082089115	8.85E-12	8.14E-09
Top2a	1335.405658	1.421287426	0.228427069	1.83E-11	1.56E-08
Cirbp	3060.816368	0.873682772	0.140204286	2.22E-11	1.77E-08
Lilrb4a	1558.564965	1.399632454	0.230511034	3.82E-11	2.54E-08
Ifi27l2a	1742.982449	1.196921756	0.195273236	3.55E-11	2.54E-08
Ddx60	2647.986453	0.937429877	0.15250758	3.68E-11	2.54E-08
Ms4a4c	3439.785048	0.887686915	0.153293067	2.94E-10	1.85E-07
Dnaja1	13054.99118	-0.507472496	0.093469896	2.88E-09	1.72E-06
Oasl2	869.2585448	1.314067398	0.252146706	8.07E-09	4.59E-06
Irgm1	6556.222225	0.528043634	0.105909578	3.15E-08	1.64E-05
Usp18	3239.100765	0.688632538	0.138900442	3.36E-08	1.68E-05
Gbp6	2556.06783	0.780411156	0.161928814	6.30E-08	2.90E-05
Irf7	4230.772344	0.613405015	0.132780915	1.77E-07	7.57E-05
Stip1	3855.705298	-0.692576806	0.153792134	2.72E-07	0.000104948
Gbp10	603.5140375	1.463571802	0.330581712	3.15E-07	0.000117478
Serpina3g	4421.57016	0.594985283	0.142428291	1.28E-06	0.000450127
Hmmr	122.3144207	3.448946253	1.001681035	1.57E-06	0.000520265
Banp	1071.20048	-1.177498809	0.298248617	2.03E-06	0.000622691
Cmpk2	3040.793258	0.64100835	0.158263658	2.18E-06	0.000650756
Fgl2	2755.562724	0.795055809	0.199147585	2.24E-06	0.000652265
Fn1	77.29749308	5.727091377	1.568819606	2.39E-06	0.000676668
Mx1	936.838965	0.940870483	0.246984736	5.59E-06	0.001421254
Arrdc3	1186.43601	-0.829958864	0.221006354	6.88E-06	0.001666724
Bub1	51.00356841	4.781031388	1.286918953	8.65E-06	0.002007875
Sgms1	2493.849922	-0.62028553	0.167676235	8.91E-06	0.002007875
Ppp2r3a	128.8639034	-2.448570203	0.67075568	8.89E-06	0.002007875
Cenpf	116.7932818	3.161347148	1.092668357	9.62E-06	0.002128539
Lgals3	3136.287894	0.550121847	0.153625288	1.47E-05	0.003200121
Dtl	233.0822416	1.77407577	0.50579133	1.53E-05	0.003254753
Kif4	112.9019861	3.169177011	1.284468771	1.64E-05	0.003436252
Cep55	123.4832335	2.587121899	0.798061359	1.87E-05	0.003814545
Cdh1	849.7499468	0.936447587	0.269298149	1.88E-05	0.003814545
Gm12250	2213.03454	0.59229958	0.168887337	1.92E-05	0.003828734

Kif11	291.6522458	1.509389512	0.444309083	2.42E-05	0.004586796
Stmn1	555.4977724	1.061133323	0.325975573	4.06E-05	0.00746975
Shcbp1	95.4041775	2.650210624	0.845503704	4.19E-05	0.007577629
Ifi27	2918.994211	0.529938294	0.167120177	6.12E-05	0.010909203
Lman2l	1025.52705	-0.767810023	0.247364303	6.84E-05	0.011679109
Plac8	2929.682151	0.541713394	0.173511474	7.00E-05	0.011785737
Kif18b	104.7855971	2.279478494	0.770884418	9.37E-05	0.014726207
Xaf1	1234.444649	0.709138553	0.237634906	0.000100573	0.015604502
Cybb	53.06502791	3.607872118	1.98383792	0.000108018	0.016131127
Mpeg1	39.88722084	4.980556374	2.131191285	0.000115238	0.01678963
Cish	1457.22481	0.616526455	0.207824597	0.000114758	0.01678963
Ccna2	185.5636013	1.63650706	0.579122181	0.000142811	0.020261979
Ankle1	24.26759784	7.055243059	3.269147474	0.000163538	0.021708765
Thsd4	101.0421971	2.51995413	0.978044012	0.000158998	0.021708765
Adgrg5	2308.854543	0.530019309	0.185346694	0.000161014	0.021708765
Slc1a4	22.59211661	6.917382004	3.273502022	0.000209484	0.027203271
Anln	70.07363788	2.91385546	1.485737249	0.000238768	0.030672718
Csf2rb	21.47924971	6.836346002	3.284328319	0.000249074	0.031656211
Ktn1	1858.168899	0.50181519	0.1961918	0.000387532	0.044517717
Gab2	35.235533	3.870348263	1.837385635	0.000422454	0.047168731

Supplementary table 2: Top 30 differentially expressed genes in memory P14 CD8 T cell clusters

Genes selectively expressed in each cluster relative to the other clusters were selected based on the Wilcoxon rank sum test. *P*-values were adjusted for multiple testing using the Bonferroni correction. The results are presented in separate pages for each cluster.

cluster	p_val	p_val_adj	avg_log2FC	pct.1	pct.2	gene
0	0	0	3.869	0.660	0.149	Gzma
	0	0	2.437	0.880	0.232	Zeb2
	0	0	2.286	0.876	0.220	Cx3cr1
	0	0	1.712	0.695	0.260	Gzmb
	0	0	1.610	0.676	0.116	S1pr5
	0	0	1.591	0.995	0.914	Lgals1
	0	0	1.532	0.718	0.232	Kcnj8
	0	0	1.210	0.440	0.056	Klrg1
	0	0	1.152	0.831	0.652	Klrc1
	0	0	1.091	0.717	0.507	Il18rap
	0	0	1.059	0.974	0.929	Ahnak
	0	0	1.057	0.715	0.439	Lgals3
	0	0	1.039	0.966	0.930	Ctsd
	0	0	1.038	0.999	0.991	Klf2
	0	0	1.038	0.574	0.363	Ccl4
	0	0	1.028	0.409	0.091	Klrb1c
	0	0	1.018	0.953	0.907	Rap1b
	0	0	0.995	1.000	0.996	Ccl5
	0	0	0.960	0.288	0.055	Klre1
	0	0	0.939	0.838	0.744	Spn
	0	0	0.927	0.454	0.162	Borcs7
	0	0	0.889	0.825	0.741	Reep5
	0	0	0.887	1.000	0.995	Nkg7
	0	0	0.879	0.984	0.964	Emp3
	0	0	0.873	0.766	0.658	Prf1
	0	0	0.873	0.875	0.823	Itgb2
	0	0	0.864	0.386	0.113	As3mt
	0	0	0.819	0.950	0.931	Cd48
	0	0	0.816	0.912	0.814	S100a4
	0	0	0.789	0.401	0.172	Bhlhe40

cluster	p_val	p_val_adj	avg_log2FC	pct.1	pct.2	gene
1	0	0	0.639	0.996	0.847	Il7r
	0	0	0.611	0.710	0.366	Kcnq1ot1
	0	0	0.488	0.815	0.446	Sidt1
	0	0	0.479	0.748	0.386	Gpr183
	0	0	0.414	0.995	0.842	Ly6e
	0	0	0.383	1.000	0.970	mt-Nd4
	0	0	0.353	1.000	0.972	Mbnl1
	0	0	0.332	1.000	0.999	Rpl12
	8.04E-307	1.31E-302	0.359	0.709	0.372	Kbtbd11
	3.03E-305	4.95E-301	0.470	0.906	0.623	BE692007
	8.97E-303	1.46E-298	0.346	1.000	0.971	mt-Nd1
	1.09E-299	1.78E-295	0.358	0.555	0.256	Actn1
	1.90E-287	3.10E-283	0.371	0.630	0.316	Slamf6
	1.86E-286	3.04E-282	0.396	0.983	0.827	mt-Nd5
	3.55E-284	5.80E-280	0.428	0.979	0.843	Xist
	2.23E-280	3.64E-276	0.423	0.520	0.244	Sell
	2.11E-278	3.44E-274	0.400	0.901	0.617	Rapgef6
	6.12E-276	9.99E-272	0.338	1.000	0.977	mt-Nd2
	2.99E-271	4.87E-267	0.463	0.914	0.661	Bcl2
	1.54E-270	2.51E-266	0.404	0.870	0.590	Satb1
	2.52E-265	4.11E-261	0.369	0.787	0.470	Ikbkb
	1.82E-239	2.97E-235	0.352	0.978	0.854	Myh9
	3.66E-235	5.97E-231	0.369	0.932	0.711	Macf1
	2.63E-228	4.29E-224	0.355	0.945	0.715	Tcf7
	1.20E-227	1.95E-223	0.422	0.934	0.709	Ctla2a
	1.74E-225	2.85E-221	0.341	0.892	0.624	Mycbp2
	2.95E-220	4.81E-216	0.332	0.826	0.528	Dgka
	1.95E-207	3.18E-203	0.333	0.840	0.561	Utrn
	5.15E-202	8.41E-198	0.338	0.914	0.659	Emb
	4.25E-184	6.94E-180	0.342	0.713	0.438	Gm26917

cluster	p_val	p_val_adj	avg_log2FC	pct.1	pct.2	gene
2	0	0	0.603	1.000	0.999	Rps19
	0	0	0.547	1.000	1.000	Rplp0
	0	0	0.521	1.000	0.999	Rpl12
	0	0	0.519	1.000	0.999	Rpsa
	0	0	0.519	1.000	0.990	Rpl36a
	0	0	0.513	1.000	0.999	Rps18
	0	0	0.510	1.000	0.999	Rps20
	0	0	0.509	1.000	1.000	Rpl13
	0	0	0.507	0.981	0.861	Gas5
	0	0	0.506	1.000	1.000	Rps15a
	0	0	0.500	1.000	0.999	Rps2
	0	0	0.496	1.000	1.000	Rps7
	0	0	0.494	1.000	1.000	Rpl32
	0	0	0.493	1.000	0.993	Eef1b2
	0	0	0.464	1.000	0.996	Rpl10a
	0	0	0.454	1.000	1.000	Rps5
	0	0	0.454	1.000	1.000	Rplp1
	0	0	0.454	1.000	1.000	Rpl17
	0	0	0.454	1.000	0.999	Rpl3
	0	0	0.454	1.000	0.999	Rpl18
	0	0	0.443	1.000	1.000	Rpl23
	0	0	0.435	1.000	0.999	Rpl39
	0	0	0.434	1.000	0.994	Rpl29
	0	0	0.433	1.000	1.000	Rpl27a
	0	0	0.428	1.000	1.000	Rps16
	0	0	0.422	1.000	1.000	Rps9
	0	0	0.419	1.000	0.999	Rps6
	2.32E-269	3.78E-265	0.455	0.993	0.854	Ltb
	7.56E-218	1.23E-213	0.429	0.581	0.358	Rgs10
	1.17E-51	1.91E-47	0.479	0.250	0.165	Dapl1

cluster	p_val	p_val_adj	avg_log2FC	pct.1	pct.2	gene
3	1.17E-238	1.91E-234	0.384	1.000	1.000	Rpl18a
	7.00E-229	1.14E-224	0.352	1.000	1.000	Rps24
	1.56E-223	2.55E-219	0.373	1.000	1.000	Rps16
	1.69E-223	2.75E-219	0.406	1.000	1.000	Rps5
	5.66E-223	9.25E-219	0.344	1.000	1.000	Rps10
	6.32E-223	1.03E-218	0.420	1.000	1.000	Rpl13
	2.06E-220	3.36E-216	0.400	1.000	0.999	Rpl18
	1.13E-219	1.85E-215	0.377	1.000	1.000	Rpl27a
	7.92E-218	1.29E-213	0.407	1.000	1.000	Rpl32
	1.79E-217	2.93E-213	0.341	1.000	1.000	Rps13
	4.59E-217	7.50E-213	0.372	1.000	1.000	Rpl17
	1.41E-216	2.31E-212	0.426	1.000	0.999	Rps18
	2.70E-212	4.41E-208	0.378	1.000	1.000	Rpl23
	2.97E-211	4.85E-207	0.357	1.000	1.000	Rps3
	2.44E-206	3.99E-202	0.390	1.000	1.000	Rps15a
	1.63E-203	2.66E-199	0.397	1.000	1.000	Rps7
	4.32E-199	7.06E-195	0.488	1.000	0.999	Rps19
	1.68E-195	2.75E-191	0.401	1.000	1.000	Rpsa
	4.71E-194	7.70E-190	0.375	1.000	1.000	Rps9
	1.59E-183	2.60E-179	0.405	1.000	1.000	Rplp0
	1.29E-181	2.10E-177	0.373	1.000	1.000	Rplp1
	4.11E-165	6.72E-161	0.390	1.000	0.997	Rpl10a
	7.23E-165	1.18E-160	0.420	1.000	0.999	Rps20
	5.29E-164	8.64E-160	0.397	1.000	0.999	Rps2
	6.65E-158	1.09E-153	0.409	1.000	0.999	Rpl12
	7.77E-149	1.27E-144	0.379	1.000	0.995	Rpl29
	4.22E-148	6.89E-144	0.347	1.000	0.999	Rpl3
	6.04E-142	9.86E-138	0.393	1.000	0.995	Eef1b2
	1.45E-86	2.37E-82	0.441	0.989	0.883	Ltb
	5.53E-54	9.04E-50	0.352	0.930	0.828	Mif

cluster	p_val	p_val_adj	avg_log2FC	pct.1	pct.2	gene
4	0	0	2.382	0.897	0.195	Isg15
	0	0	2.153	0.837	0.113	Ifit1
	0	0	1.960	0.913	0.207	Ifit3
	0	0	1.760	0.752	0.195	Slfn5
	0	0	1.180	0.619	0.076	Usp18
	0	0	1.129	0.819	0.218	Rtp4
	0	0	1.126	0.544	0.090	Isg20
	0	0	1.125	0.498	0.027	Rsad2
	0	0	0.961	0.562	0.066	Ifit3b
	3.71E-303	6.06E-299	0.902	0.159	0.007	Cxcl10
	1.05E-298	1.72E-294	1.070	0.850	0.314	Irf7
	1.78E-292	2.90E-288	0.978	0.750	0.244	Oas3
	3.58E-267	5.85E-263	1.204	0.840	0.366	Zbp1
	3.11E-265	5.09E-261	1.242	0.947	0.549	Stat1
	2.87E-244	4.69E-240	1.213	0.993	0.834	Ly6a
	2.01E-243	3.28E-239	1.517	0.950	0.637	Bst2
	4.55E-233	7.44E-229	1.078	0.992	0.839	Ifi203
	1.03E-227	1.68E-223	1.099	0.895	0.474	Trim30a
	1.73E-225	2.82E-221	1.065	0.768	0.316	Rnf213
	3.81E-225	6.22E-221	1.055	0.918	0.530	Ifi206
	4.79E-202	7.82E-198	1.005	0.724	0.294	Igtp
	1.08E-186	1.77E-182	0.958	0.821	0.425	Ifi208
	2.96E-180	4.84E-176	0.949	0.922	0.599	Slfn1
	2.14E-175	3.49E-171	1.019	0.893	0.575	Ifi209
	1.85E-157	3.02E-153	1.140	0.927	0.647	Plac8
	9.04E-155	1.48E-150	0.940	0.825	0.461	Phf11b
	1.07E-153	1.75E-149	0.939	0.798	0.427	Parp14
	2.99E-150	4.89E-146	0.969	0.966	0.785	Mndal
	4.66E-145	7.61E-141	0.972	0.954	0.815	Samhd1
	3.96E-135	6.47E-131	1.543	0.817	0.557	Ifi27l2a

cluster	p_val	p_val_adj	avg_log2FC	pct.1	pct.2	gene
5	0	0	2.111	0.482	0.011	Pclaf
	0	0	2.041	0.482	0.018	Mki67
	0	0	1.665	0.420	0.004	Birc5
	0	0	1.353	0.389	0.016	Rrm2
	0	0	1.229	0.280	0.006	Hist1h2ab
	0	0	1.164	0.528	0.016	Spc24
	0	0	1.159	0.363	0.005	Cenpf
	0	0	1.134	0.311	0.003	Ube2c
	1.40E-246	2.29E-242	2.674	0.332	0.015	Hist1h1b
	5.03E-221	8.22E-217	2.385	0.565	0.052	Top2a
	2.35E-186	3.83E-182	1.132	0.705	0.094	Lig1
	1.20E-127	1.95E-123	1.132	0.829	0.197	Mcm3
	8.82E-110	1.44E-105	2.910	0.798	0.221	Stmn1
	2.81E-95	4.59E-91	1.389	0.746	0.196	Lmnb1
	4.88E-92	7.98E-88	1.946	0.389	0.055	Hist1h2ae
	8.22E-86	1.34E-81	1.169	0.705	0.182	Smc2
	7.33E-81	1.20E-76	1.391	1.000	0.996	Ptma
	2.96E-64	4.83E-60	1.305	0.829	0.384	Dut
	2.87E-62	4.69E-58	1.447	0.933	0.501	Pcna
	5.76E-61	9.40E-57	1.295	0.824	0.344	Hmgn2
	8.07E-59	1.32E-54	1.986	0.933	0.559	Tuba1b
	8.38E-52	1.37E-47	2.206	0.984	0.872	Hmgb2
	2.59E-51	4.23E-47	1.214	0.984	0.731	Dek
	4.87E-49	7.96E-45	1.154	0.767	0.329	Atad2
	1.30E-43	2.13E-39	1.519	0.995	0.874	Tubb5
	1.29E-34	2.10E-30	3.590	0.435	0.145	Hist1h2ap
	4.13E-32	6.74E-28	1.220	0.938	0.741	H2afv
	3.47E-31	5.67E-27	1.137	0.896	0.588	Ube2s
	6.16E-18	1.01E-13	1.408	0.793	0.512	Hist1h4d
	2.77E-10	4.53E-06	1.129	0.762	0.507	Hist1h1e

cluster	p_val	p_val_adj	avg_log2FC	pct.1	pct.2	gene
6	0	0	6.088	0.543	0.014	Igkc
	0	0	5.564	0.812	0.014	Cd74
	0	0	5.513	0.435	0.002	Iglc1
	0	0	3.653	0.522	0.002	Cd79a
	0	0	3.371	0.514	0.004	Iglc2
	0	0	3.155	0.529	0.001	Ly6d
	0	0	2.610	0.507	0.002	Iglc3
	0	0	2.414	0.471	0.001	Ebf1
	0	0	2.405	0.514	0.001	Ms4a1
	0	0	2.128	0.457	0.003	Ighd
	0	0	2.074	0.152	0.001	Wfdc17
	0	0	1.969	0.435	0.010	Apoe
	0	0	1.936	0.493	0.000	Mef2c
	0	0	1.864	0.457	0.001	Fcmr
	0	0	1.776	0.442	0.001	Mzb1
	0	0	1.654	0.138	0.000	Tnfaip2
	0	0	1.639	0.442	0.003	Marcks
	6.97E-304	1.14E-299	1.730	0.167	0.002	H2-Aa
	3.98E-279	6.50E-275	2.077	0.319	0.008	Fcer1g
	1.02E-266	1.67E-262	1.883	0.232	0.004	H2-Ab1
	3.67E-221	5.99E-217	1.721	0.159	0.002	H2-Eb1
	2.55E-185	4.17E-181	2.835	0.123	0.002	Lyz2
	1.04E-130	1.70E-126	2.933	0.457	0.040	Tyrobp
	6.08E-103	9.93E-99	2.947	0.623	0.104	Cd79b
	2.75E-76	4.49E-72	2.073	0.196	0.013	Slpi
	2.91E-53	4.76E-49	4.185	0.775	0.322	Ighm
	1.17E-44	1.91E-40	1.712	0.268	0.039	Lst1
	9.85E-22	1.61E-17	1.918	0.283	0.074	Ifitm3
	6.48E-12	1.06E-07	1.938	0.203	0.064	Ifitm2
	0.0001336	1	1.863	0.362	0.233	Msrb1
