# Supplementary material for: Memory CD8 T cells are vulnerable to chronic IFN-γ signals but not to CD4 T cell deficiency in MHCII-deficient mice
Source: Nat Commun. 2024 May 28;15:4418. doi: 10.1038/s41467-024-48704-4 (PMC11133459; doi:10.1038/s41467-024-48704-4)
Supplement: Supplementary file 3 — Description of Additional Supplementary Files [file 41467_2024_48704_MOESM3_ESM.pdf]

Description of Additional Supplementary Files

Supplementary Data 1 | Gene lists used in scRNA-seq analysis

Three worksheets are provided; 1) a list of DEGs defined by bulk RNA-seq and detected by scRNA-seq; 2) a list of genes belonging to the GO categories "response to type II IFN" (GO:0034341) and "cell cycle process" (GO:0022402) and detected by scRNA-seq; 3) a list of gene signatures characterizing different memory and effector CD8 T cell subsets and detected by scRNA-seq.
