# Supplementary material for: Memory CD8 T cells are vulnerable to chronic IFN-γ signals but not to CD4 T cell deficiency in MHCII-deficient mice
Source: Nat Commun. 2024 May 28;15:4418. doi: 10.1038/s41467-024-48704-4 (PMC11133459; doi:10.1038/s41467-024-48704-4)
Supplement: Supplementary file 5 — Reporting Summary [file 41467_2024_48704_MOESM5_ESM.pdf]

nature portfolio

Corresponding author(s): Ruka Setoguchi

Last updated by author(s): 5/2/2024

Reporting Summary

Nature Portfolio wishes to improve the reproducibility of the work that we publish. This form provides structure for consistency and transparency in reporting. For further information on Nature Portfolio policies, see our Editorial Policies and the Editorial Policy Checklist.

Statistics

For all statistical analyses, confirm that the following items are present in the figure legend, table legend, main text, or Methods section.

n/a	Confirmed	
☐	☒	The exact sample size (*n*) for each experimental group/condition, given as a discrete number and unit of measurement
☐	☒	A statement on whether measurements were taken from distinct samples or whether the same sample was measured repeatedly
☐	☒	The statistical test(s) used AND whether they are one- or two-sided<br>*Only common tests should be described solely by name; describe more complex techniques in the Methods section.*
☒	☐	A description of all covariates tested
☐	☒	A description of any assumptions or corrections, such as tests of normality and adjustment for multiple comparisons
☐	☒	A full description of the statistical parameters including central tendency (e.g. means) or other basic estimates (e.g. regression coefficient) AND variation (e.g. standard deviation) or associated estimates of uncertainty (e.g. confidence intervals)
☐	☒	For null hypothesis testing, the test statistic (e.g. *F*, *t*, *r*) with confidence intervals, effect sizes, degrees of freedom and *P* value noted<br>*Give P values as exact values whenever suitable.*
☒	☐	For Bayesian analysis, information on the choice of priors and Markov chain Monte Carlo settings
☒	☐	For hierarchical and complex designs, identification of the appropriate level for tests and full reporting of outcomes
☒	☐	Estimates of effect sizes (e.g. Cohen's *d*, Pearson's *r*), indicating how they were calculated

Our web collection on statistics for biologists contains articles on many of the points above.

Software and code

Policy information about availability of computer code

Data collection	Flow cytometry: Data were collected on FACSCanto II (BD FACS Diva software version 8.0.1) or CytoFLEX S (Beckman Coulter CytExpert software version 2.3). Cell sorting was performed on FACSAria II or III using Diva software (BD ACS Diva software version 8.0.1 or 9.0.1).<br>Real-time quantitative PCR: performed on an ABI/PRISM 7000 Sequence Detection System (Applied Biosystems).<br>Cytokines and protein measurements: measured on an iMARK Microplate Reader (BIO-RAD).<br>Plasma IFN-gamma concentrations: measured on a MESO QuickPlex SQ 120MM Reader (Meso Scale Discovery).<br>Bulk RNA-seq: 100 bp paired-end reads on a Hiseq 2500 (Illumina).<br>scRNA-seq: 50 bp paired-end reads on a NovaSeq 6500 (Illumina).
Data analysis	Flow cytometry data were analyzed using FlowJo version 7.6.5 or 10.6.2 (Tree Star, Inc.).<br>Graphs were generated and statistical analyses were performed using Prism version 5 or 7 (GraphPad Software) except for bulk RNA-seq and scRNA-seq data.<br>Bulk RNA-seq: After QC and alignment to mm9 using STAR 2.5.2b, bulk RNA sequencing data (paired-end 100 bp reads) were analyzed and visualized using R version 3.6.0 and the DEseq2 and GAGE packages. Enrichment results were visualized using in-house algorithms based on d3.js.<br>scRNA-seq: After alignment, filtering, barcode counting, and molecular identifier counting using Cell Ranger v.5.0.1, scRNA sequencing data (paired-end 50 bp reads) were analyzed and visualized using Seurat 4.0.1 in R version 4.0.5.

For manuscripts utilizing custom algorithms or software that are central to the research but not yet described in published literature, software must be made available to editors and reviewers. We strongly encourage code deposition in a community repository (e.g. GitHub). See the Nature Portfolio guidelines for submitting code & software for further information.

Data

Policy information about availability of data

All manuscripts must include a data availability statement. This statement should provide the following information, where applicable:

- Accession codes, unique identifiers, or web links for publicly available datasets
- A description of any restrictions on data availability
- For clinical datasets or third party data, please ensure that the statement adheres to our policy

RNA-seq and scRNA-seq data are available at the Genomic Expression Archive (GEA) of the DNA Data Bank Japan (DDBJ) under accession numbers E-GEAD-723 (https://ddbj.nig.ac.jp/public/ddbj_database/gea/experiment/E-GEAD-000/E-GEAD-723/) and E-GEAD-724 (https://ddbj.nig.ac.jp/public/ddbj_database/gea/experiment/E-GEAD-000/E-GEAD-724/), respectively. Source codes for analysis of bulk RNA-seq and scRNA-seq are available at https://github.com/eiryo-kawakami/Setoguchi_Tcell_2024 and https://github.com/RukaSetoguchi/MHC_class_II, respectively.

Research involving human participants, their data, or biological material

Policy information about studies with human participants or human data. See also policy information about sex, gender (identity/presentation), and sexual orientation and race, ethnicity and racism.

Reporting on sex and gender	N.A.
Reporting on race, ethnicity, or other socially relevant groupings	N.A.
Population characteristics	N.A.
Recruitment	N.A.
Ethics oversight	N.A.

Note that full information on the approval of the study protocol must also be provided in the manuscript.

Field-specific reporting

Please select the one below that is the best fit for your research. If you are not sure, read the appropriate sections before making your selection.

☒ Life sciences ☐ Behavioural & social sciences ☐ Ecological, evolutionary & environmental sciences

For a reference copy of the document with all sections, see nature.com/documents/nr-reporting-summary-flat.pdf

Life sciences study design

All studies must disclose on these points even when the disclosure is negative.

Sample size	For each experiment, the sample size is indicated in the figure panels or figure legends. Sample sizes were determined based on the previous studies, including ours; PMID: 15300249, 28778586.
Data exclusions	No data was excluded.
Replication	For all experiments, at least three biological replicates were analyzed in at least two independent experiments. The number of biological replicates is indicated in each figure legend.
Randomization	Animals were assigned to groups based on genotype.
Blinding	No blinding was used as no subjective scoring methods were used.

Reporting for specific materials, systems and methods

We require information from authors about some types of materials, experimental systems and methods used in many studies. Here, indicate whether each material, system or method listed is relevant to your study. If you are not sure if a list item applies to your research, read the appropriate section before selecting a response.

Materials & experimental systems

n/a	Involved in the study
	x Antibodies
	x Eukaryotic cell lines
x	Palaeontology and archaeology
	x Animals and other organisms
x	Clinical data
x	Dual use research of concern
x	Plants

Methods

n/a	Involved in the study
x	ChIP-seq
	x Flow cytometry
x	MRI-based neuroimaging

Antibodies

Antibodies used

Antibodies for flow cytometry;

From BioLegend:
PE anti-mouse CD4 (clone RM4-4, cat No. 116005)
FITC, PE, PerCP/Cyanine5.5, or Brilliant Violet 510™ anti-mouse CD8a (clone 53-6-7, cat No.100706, 100708, 100734, 100752)
APC or PerCP/Cyanine5.5 anti-mouse CD127 (clone A7R34, cat No. 135011, 135022)
PE/Cyanine7 anti-mouse/rat/human CD27 (clone LG.3A10, cat No. 124215)
PerCP/Cyanine5.5 or APC/Cyanine7 anti-rat CD90/mouse CD90.1 (clone OX-7, cat No. 202516, 202519)
FITC or PerCP/Cyanine5.5 anti-mouse TCR Va2 (clone B20.1, cat No. 127805, 127814)
Brilliant Violet 421™ anti-mouse TCRb (clone H57-597, 109229)
PE anti-mouse/human KLRG1 (clone 2F1/KLRG1, cat No. 138407)
PE anti-mouse NK1.1 (clone PK136, cat No. 108708)
APC/Cyanine7 anti-mouse CD45.2 (clone 104, cat No. 109824)
PerCP/Cyanine5.5 anti-mouse CD8b (YTS156.7.7, cat No. 126610)

From BD Biosciences:
PE Mouse Anti-Ki-67 (clone B56, cat No. 51-36525X)
BV421 hamster anti-mouse CXCR3 (clone CXCR3-173, cat No. 562937)
APC anti-rat CD90/mouse CD90.1 (clone OX-7, cat No. 561409)
V450 anti-mouse TCRb (clone H57-597, cat No. 560706)

From eBioscience:
eFluor™ 450 anti-mouse TCR Va2 (clone B20.1, cat No. 48-5812-82)

Dilutions for these antibodies were 1/50-1/3200. Titration was performed prior to use in experiments.

Antibody for in vivo experiments;
Anti-mouse IFN-ɤ (clone XMG1.2) purchased from Bio X cell

The following hybridomas were obtained from ATCC and monoclonal antibodies were produced in-house;
Anti-mouse CD4 (clone GK1.5)
Anti-mouse CD16/32 (FcɤR) (clone 2.4G2)

Validation

Commercially available antibodies were validated by their respective manufacturers.
The reactivities of in-house produced mAbs were validated and compared to isotype control mAbs by staining mouse splenocytes with the mAbs followed by flow cytometric analysis.
The depleting activity of the GK1.5 anti-mouse CD4 mAb was confirmed by injecting the mAb into B6/J mice and staining the splenocytes with a non-competing anti-CD4 mAb (clone RM4-4).

Eukaryotic cell lines

Policy information about cell lines and Sex and Gender in Research

Cell line source(s)	Plat-E (Dr. Kitamura), 143B osteosarcoma cells (Dr. Tomura)
Authentication	Cell lines used in this study were not authenticated.
Mycoplasma contamination	Cell lines were not tested for mycoplasma contamination.
Commonly misidentified lines (See ICLAC register)	No commonly misidentified cell lines were used in this study.

Animals and other research organisms

Policy information about studies involving animals; ARRIVE guidelines recommended for reporting animal research, and Sex and Gender in Research

Laboratory animals	C57BL/6J, B6.PL-Thy1a/CyJ (stock number: 000406), B6.129S2-Cd4tm1Mak/J (CD4-/-) (stock number: 002663), and B6.129S2-H2dlAb1-Ea/J (MHCII-/-) (stock number: 003584) mice were obtained from the Jackson Laboratory. P14 TCR transgenic mice on a C57BL/6J background were kindly provided by Dr. Takashi Saito with permission of Dr. Hanspeter Pircher and crossed with B6.PL-Thy1a/CyJ mice. IFN-gamma Venus BAC transgenic reporter mice on C57BL/6J background were generated and provided by Dr. Masato Kubo.<br><br>All mice were housed at 21-25 °C room temperature, 40 to 60 % humidity, and a 12-hour light/dark cycle (7:00 am to 7:00 pm; light, 7:00 pm to 7:00 am; dark).
Wild animals	Wild animals were not used in this study.
Reporting on sex	All animals were 6-15 weeks old at the start of the experiments, and both male and female mice were used.
Field-collected samples	Field-collected samples were not used in this study.
Ethics oversight	All mice were bred, maintained, and used for experiments according to protocols approved by the Institutional Animal Care and Use Committee of RIKEN Yokohama Branch and by the Animal Care and Use Committee of The University of Tokyo.

Note that full information on the approval of the study protocol must also be provided in the manuscript.

Flow Cytometry

Plots

Confirm that:

- [x] The axis labels state the marker and fluorochrome used (e.g. CD4-FITC).
- [x] The axis scales are clearly visible. Include numbers along axes only for bottom left plot of group (a 'group' is an analysis of identical markers).
- [x] All plots are contour plots with outliers or pseudocolor plots.
- [x] A numerical value for number of cells or percentage (with statistics) is provided.

Methodology

Sample preparation	Single-cell suspensions were prepared from mouse spleens, peripheral lymph nodes, and non-lymphoid tissues. Cells were stained in PBS containing 2% FBS and 0.02 % NaN3 for 25 min on ice after incubation with anti-FcγR mAb for 5 min on ice. For Ki-67 staining, cells were stained for cell surface markers and for dead cells with Fixable Viability Dye eFluor 780, and then fixed and permeabilized with Foxp3 Staining Buffer Set (eBioscience) before staining with anti-Ki-67 mAb.
Instrument	Flow cytometry was performed using FACSCanto II (BD Biosciences) or CytoFLEX S (Beckman Coulter). Cell sorting was performed using FACSAria II or III (BD Biosciences).
Software	FACSDiva (version 8.0.1) and CytExpert (version 2.3) softwares were used for FACSCanto II and CytoFLEX S, respectively. Cell sorting was performed on FACSAria II or III using Diva software (version 8.0.1 or 9.0.1, BD Biosciences). All flow cytometry data were analyzed using FlowJo software version 7.6.5 or 10.6.2 (TreeStar).
Cell population abundance	The purity of the sorted cells was always >98% as determined by flow cytometric analysis using the same cell sorter. For all other experiments, population frequencies were noted in the corresponding figures.
Gating strategy	In all experiments, cells were first gated on a lymphocyte gate, followed by a doublet discrimination gate and a live cell gate. The gating strategy is shown in supplementary figure 7.

- [x] Tick this box to confirm that a figure exemplifying the gating strategy is provided in the Supplementary Information.
